# Occurrence of Free-living Amoebae in Nasal Swaps of Patients of Intensive Care Unit (ICU) and Critical Care Unit (CCU) and Their Surrounding Environments

**Published:** 2018-06

**Authors:** Maryam NIYYATI, Alireza NAGHAHI, Hamed BEHNIAFAR, Zohreh LASJERDI

**Affiliations:** Dept. of Medical Parasitology and Mycology, School of Medicine, Shahid Beheshti University of Medical Sciences, Tehran, Iran

**Keywords:** Immunosuppression, Hospital, Iran

## Abstract

**Background:**

The presence of potentially pathogenic Free Living Amoebae (FLA) in hospital environment could be a health hazard for high-risk patients such as immunosuppressed patients. This study was carried out to investigate the presence of potentially pathogenic FLAs in the environment and medical instruments of different hospital wards, and nasal swabs of immunosuppressed patients of a hospital in Tehran, Iran.

**Methods:**

In this cross-sectional study, 60 environmental (26 samples) and nasal swab (34 samples) samples were collected between Dec 2015 and Feb 2016. The samples were assessed using culturing, staining and morphological methods based on page key. To decrease the bacterial and fungal contamination and better identification of FLAs, cloning was performed.

**Results:**

Overall, 17 (28%) samples, including 13 environmental samples and 4 nasal swabs samples, were found positive for FLAs. The most frequent amoebae were *Acanthamoeba* spp. and two plates had mix contamination of *Acanthamoeba* spp. and Vahlkampfiids/*Vermamoeba.* Overall, *Acanthamoeba* species (58%), Vahlkampfiids (26%) and *V. vermiformis* (15%) were identified in clinical and environmental samples.

**Conclusion:**

The occurrence of these FLAs in environmental and clinical samples of hospital may threat health status of patients directly, particularly in immunosuppressed patients, and can transmit other pathogens. Thus, the increasing awareness of clinical setting staffs about FLAs and improvement of disinfection methods in hospitals is needed.

## Introduction

Free-living amoebae (FLA) are the opportunistic, amphizoic and ubiquitous protozoan. FLAs have global distribution and were isolated from various resources such as water, soil, air, wastewater and other environmental resources, and clinical samples (1). Some genera of FLAs such as *Naegleria, Acanthamoeba, Balamuthia*, and *Sappinia* are medically important and are causative agents of opportunistic and non-opportunistic infections in humans (1–3). Recently, researchers have reported *Vahlkampfia, Paravahlkamfia,* and *Vermamoeba* responsible for FLA-related diseases (4–7).

There is an increasing trend regarding *Acanthamoeba* keratitis (AK) in Iran (8). However, no researches have been done to detect FLA-encephalitis in this region. There is a single report presenting *N. fowleri* occurrence in a 5-month infant in Iran. This patient recovered using drug therapy (9).

The immunological status of the host is the main risk factor for developing *Acanthamoeba* encephalitis and the raised number of immunodeficient hosts has resulted in an increase of the infection incidence (10). The majority of GAE cases have occurred in immune-suppressed patients such as HIV, graft receivers, steroid users, leukemia, and cirrhotic and hepatitis patients (10, 11). The main routes of GAE acquisition are inhalation of cysts and entrance of the agent through skin wound (2). The presence of free-living amoeba cysts in hospital environment is a health threat. Moreover, these amoebas can transport and transmit pathogenic bacteria (12). Cyst of FLA including *Acanthamoeba* spp. are very resistant to disinfect-ants and they can resist harsh environment (13). *Acanthamoeba* could present in oxygen mask of an isolated room (14).

There is limited data regarding the presence of free-living amoeba in both hospitalized patients and their clinical setting environment. Indeed, few studies have been conducted in Iran regarding the occurrence of *Acanthamoeba* spp. in cancer patients and immunosuppressed patients (15).

Intensive care unit and critical care unit admit patients with severe condition and any contamination of these wards to pathogenic microorganisms could be a healthy treat for patients. Occurrence of FLAs in the medical instruments and hospital environment can be a risk factor to the health of patients (16).

Therefore, the main aim of the present study was the determination of the occurrence of FLAs in immune-suppressed patients and in dust from ICU and CCU hospital ward in Tehran, Iran, using culturing and microscopic methods. To the best of our knowledge, this is the first study that investigates the presence of FLAs in environmental sources of hospital wards, medical instruments, and patients, simultaneously.

## Materials and Methods

### Samples

In this cross-sectional study, 60 samples were collected between Dec 2015 and Feb 2016, from intensive care units and critical care units of a hospital in Tehran, Iran. In this study, various locations were selected including surgical, general and heart ICU and CCU randomly for taking samples, and sterile swabs were used for sample collection. All samples were transferred within 24 h to the Protozoology Laboratory of Shahid Beheshti University of Medical Science, Tehran, Iran.

### Clinical samples

Overall, 34 nasal swabs were collected from hospitalized patients. Due to corticosteroid therapy, all of the selected patients were immune-suppressed. Selected patients were hospitalized in CCU (16), surgical ICU (5), general ICU (8) and open heart surgery ICU (5) wards.

All patients were informed regarding the study procedure and they were all satisfied to participate in the present research. Ethics Committee of the university approved the study.

### Environmental samples

26 dust samples were collected from CCU (16), surgical ICU (4), general ICU (4) and open heart surgery ICU (2) wards. Samples were taken from medical instruments, central air conditioners, windows, and doors. Because of isolation in ICU wards, we collected our samples only from central air conditioners.

### Isolation and identification of FLAs based on page key

All of environmental swabs were washed in approximately 200 mL sterile water. Water samples were then filtered through cellulose nitrate membranes (pore size, 1.6 μm) (17). Central part of each membrane was incubated on 1.5% non-nutrient agar (NNA) plate overlaid with a mono-layer of heat-inactivated *Escherichia coli* (18). Nasal swabs were cultured on plates directly after sampling. The plated were then sealed and incubated at 28 °C. One week after cultivation the plates were investigated (using 100X magnification of microscope) daily for out-growth of FLAs up to two months. Positive samples were investigated using page key. Morphological characteristics of trophozoites and cysts were used to identify FLAs according to page key (19). To decrease the bacterial and fungal contamination and better identification of FLAs, cloning was performed. To this end, a small part of agar containing amoebae cysts was cut and placed in fresh medium. This procedure was done to achieve a pure plate.

## Results

**Fig. 1:**
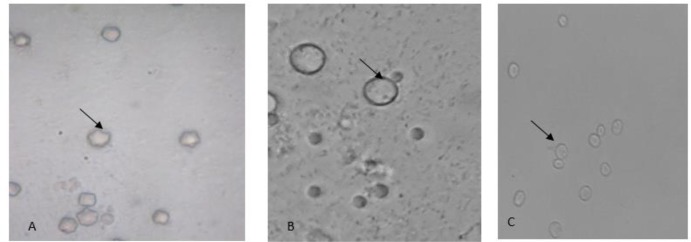
A: *Acanthamoeba* cysts (× 100), B: Vahlkampfiids cyst (×1000), C: *Vermamoeba* cysts (×1000)

Overall, 17 (28%) samples out of 60 samples were found positive for FLAs and 19 isolates were detected. Thirteen dust samples (50%) from ICU and CCU wards and 4 nasal swap samples (12%) from severe immunosuppressed patients were positive for outgrowth of free-living amoebae (Tables 1 and 2). Medical instrument of CCU wards was the most contaminated source. Accordingly, nasal swaps of hospitalized patients in the same CCU wards were also showed highest contaminated sources. *Acanthamoeba* spp. was detected in 30.7% and 8.8% of dust and nasal swabs, respectively according to double-walled cyst and flat shaped trophozoites (Fig. 1). According to page key, all isolates belonged to morphological group 2. Vahlkampfiids were detected by characteristic round cysts measuring 10 μ and elongated shape trophozoites (Fig. 1). *V. vermiformis* were also characterized using its wormy shape trophozoites and round cysts (Fig. 1). In this study, the most frequent amoeba was *Acanthamoeba* spp. and two plates had mix contamination of *Acanthamoeba* spp. and Vahlkampfiids/*Vermamoeba* (Table 3).

**Table 1:** Data from isolated FLAs from environmental sources in CCU and ICU wards

***Collection site***	***No. of samples***	***No. of positive samples (%)***	***Isolated FLA***
CCU	16	11 (69)	*Acanthamoeba* spp. Vahlkampfiids *Vermamoeba vermiformis*
Surgical ICU	4	1 (25)	*Acanthamoeba* spp.
General ICU	4	1 (25)	*Acanthamoeba* spp.
Open heart ICU	2	00 (00)	-
Total	26	13 (50)	*Acanthamoeba* spp. Vahlkampfiids *Vermamoeba vermiformis*

**Table 2:** Data from FLAs isolated from nasal swaps of hospitalized patients in CCU and ICU wards

***Collection site***	***No. of samples***	***No. of positive sample (%)***	***Isolate FLA***
CCU	16	4 (25)	*Acanthamoeba* spp. Vahlkampfiids *Vermamoeba vermiformis*
Surgical ICU	5	00 (00)	-
General ICU	8	00 (00)	-
Open heart ICU	5	00 (00)	-
Total	34	4 (12)	*Acanthamoeba* spp. Vahlkampfiids *Vermamoeba vermiformis*

**Table 3:** Frequency of isolated FLA from patients and hospital environment

***Isolated FLA***	***No.***	***Percentage***
*Acanthamoeba* spp.	11	58
Vahlkampfiids	5	26
*Vermamoeba vermiformis*	3	15

## Discussion

This study was the first investigations of the occurrence of FLAs in dust from ICU and CCU wards and hospitalized immune-suppressed patients (high-risk people) simultaneously in Iran. Detected FLAs belonged to three genera, including *Acanthamoeba* (11), Vahlkampfiids (5) and *Vermamoeba* (3). FLAs were isolated from various environmental sources and patients in Iran (20–23). Occurrence of FLAs was reported 52.9% and 42.86% in dust and biofilm samples of immunodeficiency and ophthalmology wards of hospital in Iran, respectively (14, 24). There are various study on occurrence of FLAs in therapeutic pools and water system of hospital (7, 16, 25–27), and some studies were conducted to investigate FLAs contamination of other sources in hospital worldwide (14, 28, 29).

Dust was previously reported as potential source for *Acanthamoeba*, *Balamuthia* and Vahlkampfiid amoebae in Iran (14, 30, 31). Due to heavy air pollution and airborne dust in Tehran, cyst form of FLAs can easily be transferred to visitors and patients of hospital wards (28, 32, 33).

In addition to dust samples, the present study evaluated the presence of FLAs in nasal swabs of hospitalized patients. In Peru, 21 (28.4%) samples out of 74 nasal swabs positive for *Acanthamoeba* species in healthy individuals were found (34). However, no study was conducted to evaluate the presence of all of medically important FLAs in nasal swabs of patients or healthy individuals. As expected, among three detected amoebas *Acanthamoeba* belonging to group 2 has the highest occurrence in both of the dust and nasal swabs. This can be due to high resistance of *Acanthamoeba* cysts to usual chlorine-based disinfectants used in the most of the Tehran hospitals (10, 28).

In this study, all of the nasal swabs were taken from patients’ immune-suppressed by corticosteroid therapy. Deficient or suppressed immune system is an important risk factor for most of the FLAs induced infections, such as GAE.

## Conclusion

The high occurrence of FLAs in environmental samples of the selected hospital wards, and it may result in life-threating infections. On the other hand, all of the FLAs detected in dust samples, were isolated from CCU patients too, thus dust may be source of infection for patients. Increase awareness of hospital staff about FLAs, improvement of disinfection methods and using efficient disinfectants in hospital, particularly in wards which immunodeficient or immune-suppressed patients hospitalized is necessary.

## Ethical considerations

Ethical issues (Including plagiarism, informed consent, misconduct, data fabrication and/or falsification, double publication and/or submission, redundancy, etc.) have been completely observed by the authors.
